# Tunneling induced two-dimensional phase grating in a quantum well nanostructure via third and fifth orders of susceptibility

**DOI:** 10.1038/s41598-020-64255-2

**Published:** 2020-04-30

**Authors:** Azar Vafafard, Mostafa Sahrai, Hamid Reza Hamedi, Seyyed Hossein Asadpour

**Affiliations:** 1 Departement of Physics, University of Tabriz, Tabriz, Iran; 2 Institute of Theoretical Physics and Astronomy, Vilnius University, Saulėtekio 3 LT-10257, Vilnius, Lithuania; 3 Young Researchers and Elite Club, Central Tehran Brach, Islamic Azad University, Tehran, Iran

**Keywords:** Optics and photonics, Physics

## Abstract

We study the nonlinear optical properties in an asymmetric double AlGaAs/GaAs quantum well nanostructure by using an external control field and resonant tunneling effects. It is found that the resonant tunneling can modulate the third-order and fifth-order of susceptibilities via detuning frequency of coupling light. In presence of the resonant tunneling and when the coupling light is in resonance with the corresponding transition, the real parts of third-order and fifth-order susceptibilities are enhanced which are accompanied by nonlinear absorption. However, in off-resonance of coupling light, real parts of third-order and fifth-order susceptibilities enhance while the nonlinear absorption vanishes. We investigate also the two-dimensional electromagnetically induced grating (2D-EIG) of the weak probe light by modulating the third-order and fifth-order susceptibilities. In resonance of coupling light, both amplitude and phase grating are formed in the medium due to enhancement of third-order and fifth-order probe absorption and dispersion. When the coupling light is out of resonance, most of probe energy is transferred from zero-order to higher-order directions due to resonant tunneling effect. The efficiency of phase grating for third-order of susceptibility is higher than phase grating for fifth-order susceptibility. Our proposed model may be useful for optical switching and optical sensors based on semiconductor nanostructures.

## Introduction

It is known that the electromagnetically induced transparency (EIT)^1^ leads to many appealing optical effects in quantum and nonlinear optics^2–8^. An important and favorable phenomenon arising from interaction of a standing-wave field with the EIT medium is known as electromagnetically induced grating (EIG)^9^. The EIG is due to a periodic spatial modulation of absorption and dispersion of an atom. Many proposals have been suggested to investigate the EIG in various media^10–15^. The coherent control of the EIG patterns via spontaneous emission has been studied by Xie *et al*.^16^. Considering a three-level atomic system in the Λ configuration with two closely-lying lower levels and in the presence of spontaneously generated coherence (SGC), it has been found that the diffraction efficiency of phase grating can be enhanced due to the presence of SGC. In another work by Gao^17^, the SGC effect on Kerr nonlinearity and EIG of four-level atomic system were investigated. It was shown that as a result of the SGC, it is possible to achieve an enhanced nonlinear refraction with zero linear absorption. The diffraction efficiency of weak probe light can transfer from zero-order to higher-order components. The effect of SGC on EIG pattern in a four-level N-type atomic system was also investigated^18^. It was shown that the SGC can adjust the diffraction efficiency of high order patterns in the presence of incoherent pumping field. The relative phase between applied fields due to the presence of SGC can influence the diffraction efficiency of the phase grating. Two dimensional electromagnetically induced grating (2D EIG) has been also studied in a four-level double Λ-type atomic system via incoherent pumping field^19^. It has been shown that by applying a standing-wave beam, the higher order Fraunhofer diffraction can be obtained for a probe light due to the modulation of the refractive index. Recently, the discreet diffraction patterns established via optically induced atomic lattices have been also experimentally demonstrated^20^.

ElGs with their adjustable optical properties are proper candidates for all the areas which require the diffraction grating. For instance, the structure of photonic band gaps can be improved by optically induced lattice^21^. EIG also makes it possible to store the propagated light through an atomic medium^22^. Construction of all-optical beam splitting and fanning using EIG have been presented in^23^. Diffraction of light via EIGs can also be employed to form the electromagnetically induced Talbot effect which is essential for imaging of two-dimensional ultra-cold atoms^24^. We recall that recently, the study of nonlinear susceptibilities and their effects on the behavior of quantum systems has attracted a great deal of attention^25,26^. A new scheme of multi-component vector solitons based on EIG have been introduced^27^. The generation of two-dimensional surface solitons of a four-wave mixing signal has been also experimentally demonstrated in an optically induced atomic lattice^28^.

On the other hand, optical properties of semiconductor quantum wells (SQWs) or quantum dots (SQDs) are well studied due to their large electric dipole, high nonlinearity and flexible designed^29–34^. The theory of quantum coherence phenomena in SQDs has been discussed by Chow *et al*.^35^. It was shown that gain without population inversion (GWI), EIT and enhancement in refractive index can be obtained for dephasing rate at room temperature. Several other studies have been carried out afterwards, on optical properties of SQWs and SQWs and based on quantum coherence and interference effect^36–41^. For example, Li *et al*.^37^ investigated the optical bistability and multistability in unidirectional ring cavity with SQWs. They found that the bistable threshold can be adjusted by using of intense laser field. The effect of tunneling induced quantum interference on optical properties of SQW nanostructure has also been studied recently^42–46^. Yang *et al*.^42^, studied the tunneling induced giant Goos-Hanchen shift in SQW. It is realized by using an external control light beam and strength of resonant tunneling, the GH shifts of transmitted and reflected light beams from a cavity with a four-level SQW system can be manipulated. Peng *et al*. investigated nonlinear properties of a coupled QD system via resonant tunneling effect. It was realized that the linear absorption converts to the gain when the self-Kerr nonlinearity of the system increases. EIGs have been also offered to produce a two-port all-optical switch as illustrated in an experiment with a three-level $\varLambda$-type system^47^. The standing wave used in such an EIG setup is made of two counter-propagating control fields. In the absence of the backward-propagating control field, the probe field is transmitted through the medium without reflection as a result of EIT in the forward direction. As soon as the counter-propagating control field is switched on, the probe field is reflected in the opposite direction as a consequence of EIG. Such a switch is accessible by tuning the intensity of the counter-propagating control field.

In this paper, we propose a model for obtaining a 2D-EIG in a SQW nanostructure by using a coupling laser field with two-dimensional standing-wave pattern. Expanding the susceptibility of the medium into higher orders of the coupling light, we investigate the effect of resonant tunneling on the third-order and fifth-order susceptibilities in resonance and off-resonance of the coupling light. It is found that the third-order and fifth-order susceptibilities can be modulated by adjusting the controllable parameters of the system. The 2D-EIG patterns of the weak probe light are then analyzed by using the third-order and fifth-order nonlinearities. We realize that the energy of probe light can be transferred from zero-order to higher-order direction by modulating both third- order and fifth-orders susceptibilities. However, the efficiency of two- dimensional phase grating for third-order susceptibility is higher than fifth-order susceptibility. In the presence of Fano interference in our proposed EIG scheme, competition between the third-order and fifth-order nonlinear susceptibilities enables the switching between amplitude and phase gratings resulting in increasing the efficiency of diffraction.

## Model and Equations

**Figure 1:**
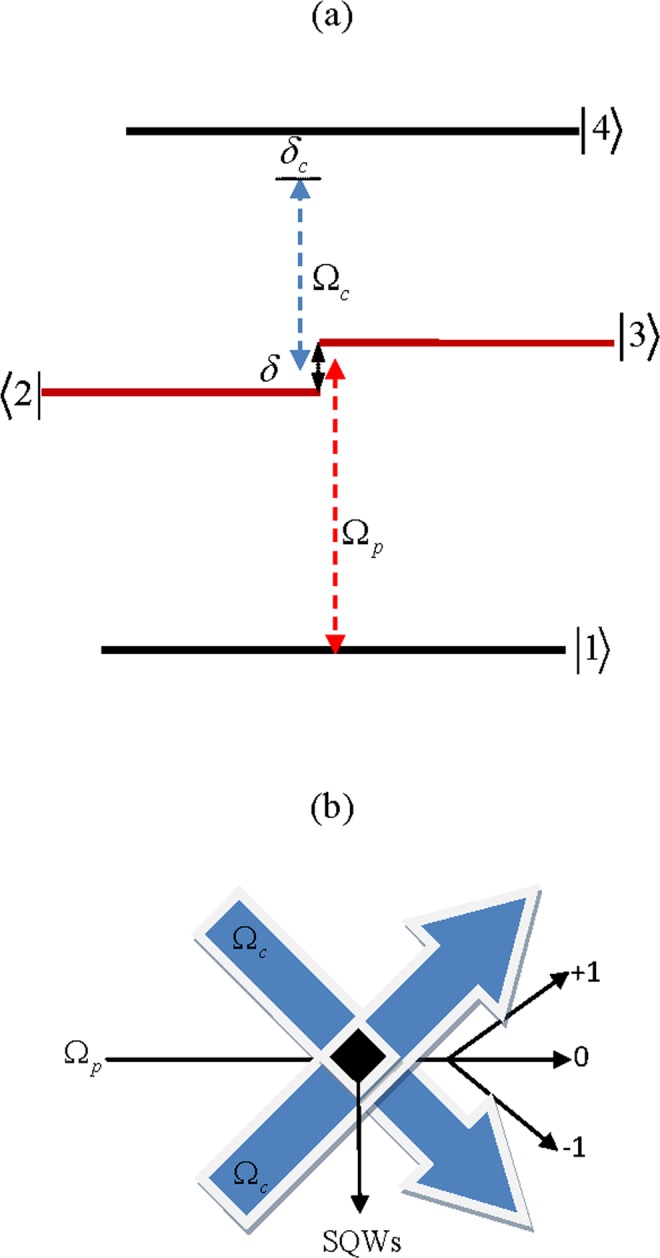
(**a**) Four-level semiconductor quantum well system interacted with a weak probe light and a coupling field with standing-wave pattern. (**b**) The probe and coupling fields propagating through SQWs.

The schematics of four sub-band SQWs nanostructure with relevant conduction band levels are presented in Fig. 1(a). The energies of the ground state and first excited state are ${E}_{1}=46.7\,meV$ and ${E}_{4}=296.3\,meV$, respectively^42^. By using resonant tunneling two intermediate states $|2\rangle$ and $|3\rangle$ with energies ${E}_{2}=174.8\,meV$ and ${E}_{3}=183.5\,meV$ can be obtained, respectively^42^. The weak probe light couples the ground level $|1\rangle$ to intermediate states $|2\rangle ,\,|3\rangle$ via a weak probe light with frequency ${\omega }_{p}$ and Rabi-frequency ${\Omega }_{p}$. The control light ${\Omega }_{{\rm{c}}}$ with frequency ${\omega }_{c}$ couples the intermediate states $|2\rangle ,\,|3\rangle$ to the excited state $|4\rangle$. Under the rotating-wave and dipole approximation, the equations of motion of the amplitude are given as follows:

1 $$\begin{array}{c}{\dot{c}}_{1}=ig{\Omega }_{p}^{\ast }{c}_{2}+i{\Omega }_{p}^{\ast }{c}_{3},\\ {\dot{c}}_{2}=i(i{\gamma }_{2}-{\delta }_{p}+\delta ){c}_{2}+ig{\Omega }_{p}{c}_{1}+if{\Omega }_{c}^{\ast }{c}_{4}+\eta {c}_{3},\\ {\dot{c}}_{3}=i(i{\gamma }_{3}-{\delta }_{p}-\delta ){c}_{3}+i{\Omega }_{p}{c}_{1}+i{\Omega }_{c}^{\ast }{c}_{4}+\eta {c}_{2},\\ {\dot{c}}_{4}=i(i{\gamma }_{4}-{\delta }_{p}-{\delta }_{c}){c}_{4}+if{\Omega }_{c}{c}_{2}+i{\Omega }_{c}{c}_{3}.\end{array}$$

The parameters $${\delta }_{p}=(\frac{{E}_{3}+{E}_{1}}{2}-{E}_{1})-{\omega }_{p},\,{\delta }_{c}=({E}_{4}-\frac{{E}_{3}+{E}_{1}}{2})-{\omega }_{c}$$ show detuning for the probe and coupling lights, respectively. Here, $$2\delta =({E}_{3}-{E}_{2})$$ corresponds to the energy splitting between intermediate state $$|2\rangle $$$$|3\rangle $$, while $$g={\mu }_{21}/{\mu }_{31}$$ and $$f={\mu }_{24}/{\mu }_{34}$$ show the ratio between the relevant transition dipole moments. The total decay rates are shown by $${\gamma }_{i}(i=1-4)={\gamma }_{il}+{\gamma }_{id}$$, where $${\gamma }_{il}$$ shows the population decay rates and $${\gamma }_{id}$$ denotes the dephasing rates, respectively. The term $$\eta =\sqrt{{\gamma }_{2l}.{\gamma }_{3l}}$$ shows a cross-coupling term among levels $$|2\rangle $$ and $$|3\rangle $$ due to tunneling effect^42^. By solving Eq. (1) in the steady-state regime and by using the polarization of the medium $$P={\varepsilon }_{0}{\chi }_{p}{E}_{p}=2N({\mu }_{21}{c}_{2}{c}_{1}^{\ast }+{\mu }_{31}{c}_{3}{c}_{1}^{\ast })$$, the susceptibility of the medium reads

2a $${\chi }_{p}=\frac{N{\mu }_{31}^{2}}{{\varepsilon }_{0}\hslash }\chi ,$$

2b $$\chi =-\frac{[{\Gamma }_{4}A-{(f-g)}^{2}{\Omega }_{c}^{2}]}{{\Gamma }_{4}B-{\Omega }_{c}^{2}C}$$

where $\,A={f}^{2}{\Gamma }_{2}+{\Gamma }_{3}-2if\eta ,\,B={\Gamma }_{2}{\Gamma }_{3}+{\eta }^{2},$ $C={g}^{2}{\Gamma }_{2}+{\Gamma }_{3}-2ig\eta ,\,{\Gamma }_{2}={\Delta }_{p}-\delta +i{\gamma }_{2},\,{\Gamma }_{3}={\Delta }_{p}+\delta +i{\gamma }_{3}$, and $\Gamma ={\Delta }_{{\rm{p}}}+{\Delta }_{{\rm{t}}}+{\rm{i}}{\gamma }_{4}$. In what follows, we obtain the third-order and fifth-order susceptibilities between the coupling and the probe fields, by expanding the probe susceptibility $\chi ({\omega }_{p})$ into second and fourth orders of ${\Omega }_{c}$ by using the Maclaurin formula:

3 $$\chi =[{\chi }^{(1)}+{\chi }^{(3)}{\Omega }_{c}^{2}+{\chi }^{(5)}{\Omega }_{c}^{4}],$$

where ${\chi }^{(1)},{\chi }^{(3)}$ and ${\chi }^{(5)}$ stand for the first, third- and fifth-order susceptibilities, respectively, defined by

4a $${\chi }^{(1)}\propto -\frac{A}{B}$$

4b $${\chi }^{(3)}\propto \frac{(\,-\,AC+B{(f-g)}^{2})}{{B}^{2}{\Gamma }_{4}},$$

4c $${\chi }^{(5)}\propto \frac{C(\,-\,AC+B{(f-g)}^{2})}{{B}^{3}{\Gamma }_{4}^{2}}.$$

Equation (3) illustrates that the total susceptibility of the medium is related to the intensity of coupling light via third-order and fifth-order susceptibilities. By employing a coherent coupling field with standing-wave pattern ${\Omega }_{c}={\Omega }_{c0}[\,\sin (\pi x/{\Lambda }_{x})+\,\sin (\pi y/{\Lambda }_{y})]$ and space period ${\Lambda }_{x},\,{\Lambda }_{y}$, the diffraction pattern of the probe light through the medium can be obtained (Fig. 1(b)) by using the Maxwell equation under the slowly varying envelope approximation and in steady-state regime:

5 $$\frac{\partial {E}_{p}}{\partial z}=i\frac{\pi }{{\varepsilon }_{0}{\lambda }_{p}}{P}_{p},\,{P}_{p}={\varepsilon }_{0}{\chi }_{p}{E}_{p},$$

Parameter ${\lambda }_{p}$ corresponds to the wavelength of the probe light. The transmission function for an interaction length L of the two-dimensional grating is then given by:

6 $$T(x,y)={e}^{-\text{Im}({\chi }^{(1)}+{\chi }^{(3)}{\Omega }_{c}^{2}+{\chi }^{(5)}{\Omega }_{c}^{4})L}{e}^{i\mathrm{Re}({\chi }^{(1)}+{\chi }^{(3)}{\Omega }_{c}^{2}+{\chi }^{(5)}{\Omega }_{c}^{4})L}$$

By using the Fourier transformation of the transmission function T(x, y), we obtain Fraunhofer diffraction equation

7 $${I}_{p}({\theta }_{x},{\theta }_{y})={|E({\theta }_{x},{\theta }_{y})|}^{2}\frac{{\sin }^{2}(M\pi {\Lambda }_{x}\,\sin \,{\theta }_{x}/{\lambda }_{p}){\sin }^{2}(N\pi {\Lambda }_{y}\,\sin \,{\theta }_{y}/{\lambda }_{p})}{{M}^{2}{N}^{2}{\sin }^{2}(\pi {\Lambda }_{x}\,\sin \,{\theta }_{x}/{\lambda }_{p}){\sin }^{2}(\pi {\Lambda }_{y}\,\sin \,{\theta }_{y}/{\lambda }_{p})},$$

with

8 $$E({\theta }_{x},{\theta }_{y})={\int }_{0}^{1}\exp (\,-\,i2\pi x{\Lambda }_{x}\,\sin \,{\theta }_{x}/{\lambda }_{p})dx\times {\int }_{0}^{1}T(x,y)\exp (\,-\,i2\pi x{\Lambda }_{y}\,\sin \,{\theta }_{y}/{\lambda }_{p})dy,$$

Where ${\theta }_{x}$ and ${\theta }_{y}$ show the diffraction angle with respect to the z-direction. The (m, n)-th order diffraction angle is determined by the grating equation $\sin \,{\theta }_{x}=m{\lambda }_{p}/{\Lambda }_{x}$ and $\sin \,{\theta }_{y}=n{\lambda }_{p}/{\Lambda }_{y}$, where m and n are the spatial period numbers of the atomic grating.

## Results and Discussion

**Figure 2:**
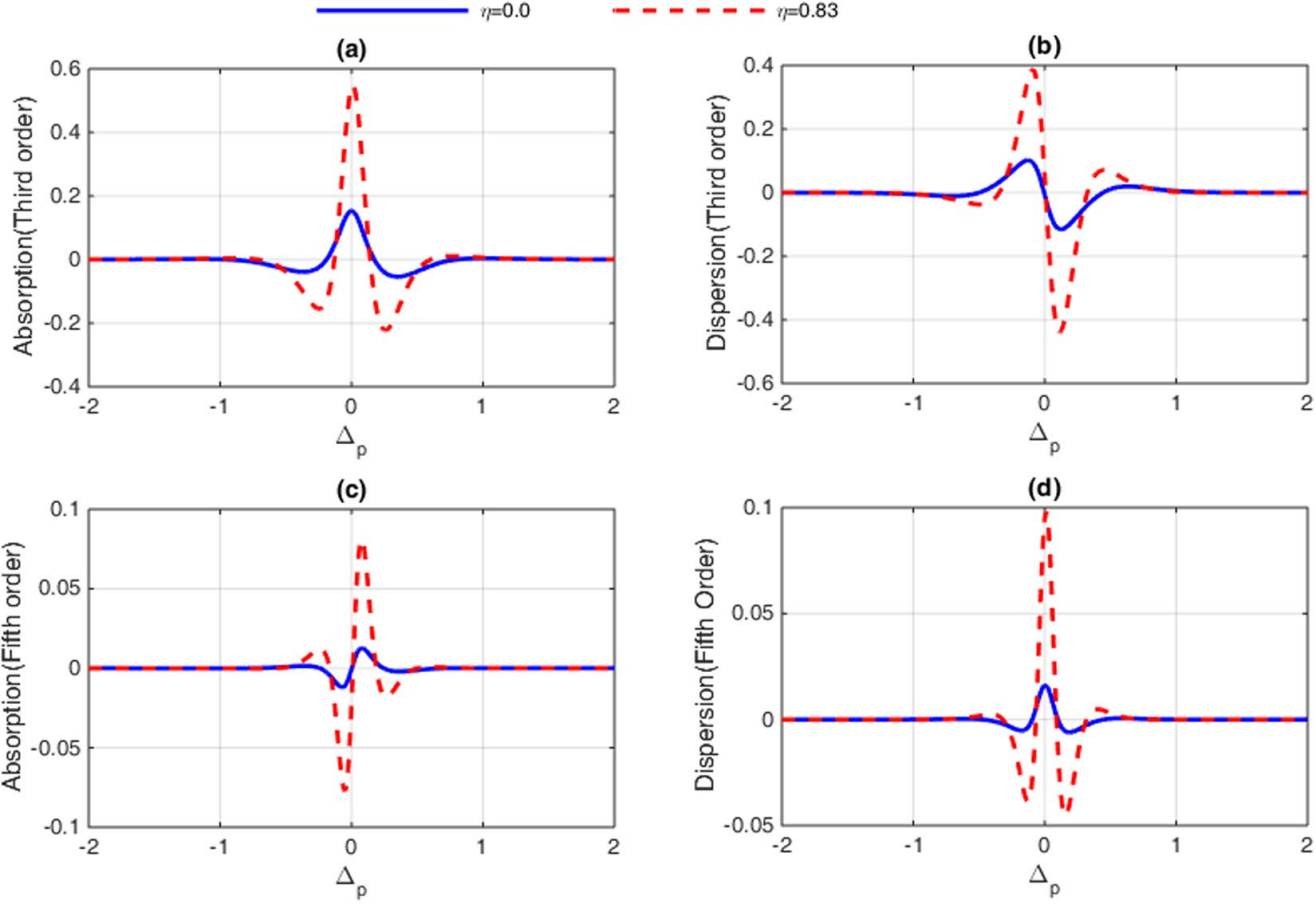
Absorption (**a,c**) and dispersion (**b,d**) of third- and fifth-order of susceptibility versus probe detuning. Solid line corresponds to absence of Fano interference and dashed line corresponds in presence of Fano interference. The selected parameters are ${\delta }_{c}=0,\,\delta =2\gamma$, ${\Omega }_{c}=0.65\gamma ,\,$ and ${\gamma }_{2l}=0.84\gamma ,\,{\gamma }_{3l}=1\gamma ,$ ${\gamma }_{4l}=0.19\gamma ,{\gamma }_{4d}=0.12\gamma$. Our calculation is based on MATLA R2014b software. https://www.mathworks.com/.

**Figure 3:**
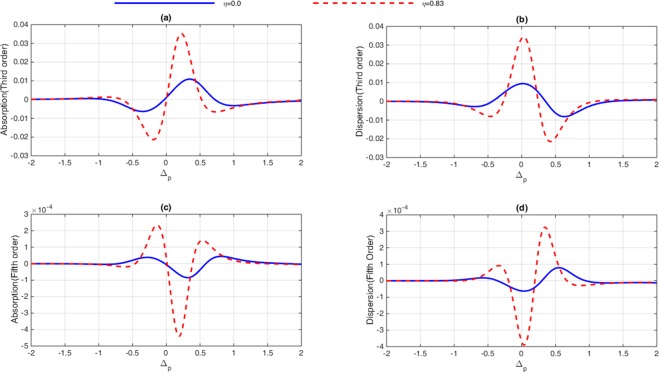
Absorption (**a,c**) and dispersion (**b,d**) of third- and fifth-order of susceptibility versus probe detuning. Solid line corresponds to absence of Fano interference and dashed line corresponds in presence of Fano interference. The selected parameters are ${\delta }_{c}=-\,6\gamma ,\,\delta =2\gamma$, ${\Omega }_{c}=0.65\gamma ,\,$ and ${\gamma }_{2l}=0.84\gamma ,\,{\gamma }_{3l}=1\gamma ,$ ${\gamma }_{4l}=0.19\gamma ,{\gamma }_{4d}=0.12\gamma$. Our calculation is based on MATLA R2014b software. https://www.mathworks.com/.

**Figure 4:**
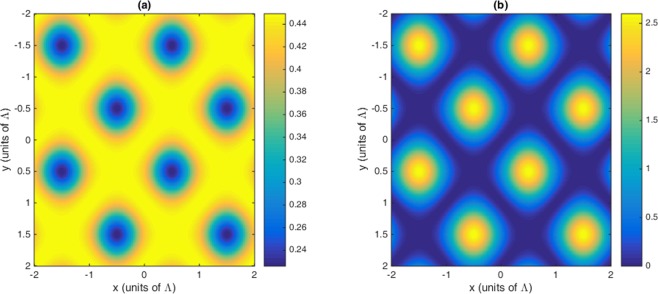
Amplitude (**a**) and phase (**b**) of total susceptibility versus x and y in the absence of Fano interference. The selected parameters are ${\Omega }_{c}=0.65\gamma ,\,{\gamma }_{2d}=0.32\gamma ,{\gamma }_{3d}=0.38\gamma ,{\gamma }_{2l}=0.84\gamma ,\,{\gamma }_{3l}=1\gamma ,{\gamma }_{4l}=0.19\gamma ,$ ${\gamma }_{4d}=0.12\gamma ,{\delta }_{c}=-6\gamma ,\,\delta =2\gamma$. Our calculation is based on MATLA R2014b software. https://www.mathworks.com/.

**Figure 5:**
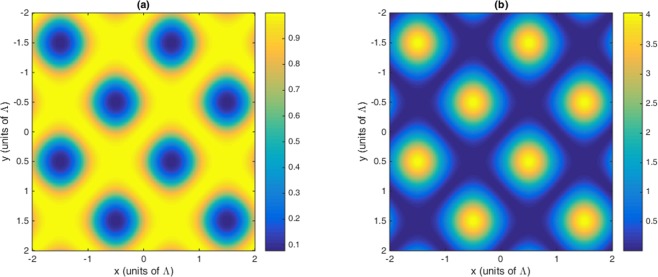
Amplitude (**a**) and phase (**b**) of total susceptibility versus x and y in the presence of Fano interference. The selected parameters are ${\Omega }_{c}=0.65\gamma ,\,{\gamma }_{2d}=0.32\gamma ,\,{\gamma }_{3d}=0.38\gamma ,\,{\gamma }_{2l}=0.84\gamma ,\,{\gamma }_{3l}=1\gamma ,\,{\gamma }_{4l}=0.19\gamma ,$ ${\gamma }_{4d}=0.12\gamma ,{\delta }_{c}=-6\gamma ,\,\delta =2\gamma$. Our calculation is based on MATLA R2014b software. https://www.mathworks.com/.

**Figure 6:**
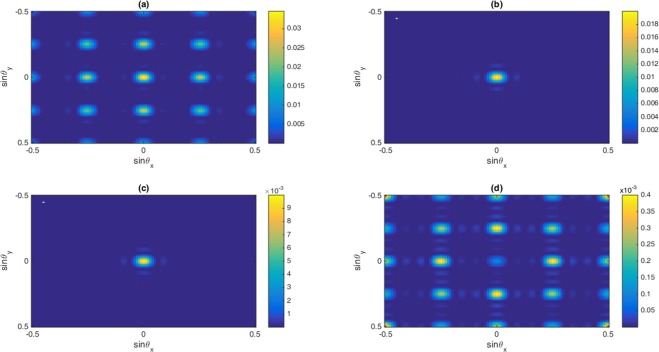
The Normalized Fraunhofer diffraction intensity versus $\sin \,{\theta }_{x}$ and $\sin \,{\theta }_{y}$ in the absence of Fano interference for (**a**) total susceptibility, (**b**) first-, (**c**) third- and (**d**) fifth-order of susceptibility. The selected parameters are ${\Omega }_{c}=0.65\gamma ,{\gamma }_{2d}=0.32\gamma ,{\gamma }_{3d}=0.38\gamma ,{\gamma }_{2l}=0.84\gamma ,\,{\gamma }_{3l}=1\gamma ,\,{\gamma }_{4l}=0.19\gamma ,{\gamma }_{4d}=0.12\gamma ,$ ${\delta }_{c}=0,\,\delta =2\gamma$. Our calculation is based on MATLA R2014b software. https://www.mathworks.com/.

In this section, the role of Fano interference is considered in the study of optical properties of nonlinear susceptibilities as well as the 2D-EIG in an SQW. It is known that the term $\eta =\sqrt{{\gamma }_{2l}.{\gamma }_{3l}}$ represents the cross-coupling term between levels $|2\rangle$ and $|3\rangle$ related to the tunneling effect from the electronic continuum^42^. Moreover, the parameter $p=\eta /\sqrt{{\gamma }_{2}.{\gamma }_{3}}$ denotes the strength of Fano-type interference induced by strong tunneling. In the absence (presence) of Fano interference, we consider $p=0(p=1)$, respectively. In Fig. 2, we display the absorption and dispersion of third-order (a, b) and the fifth-order (c, d) nonlinear susceptibilities versus probe detuning in the absence and presence of Fano interference. Here, we consider that the coupling light is in resonance with the corresponding transition i.e., ${\delta }_{c}=0$. The solid line is plotted for $\eta =0$ while the dashed line is depicted for $\eta =0.83$. In the absence of Fano interference, the third-order nonlinearity shows large absorption accompanied by zero cross-Kerr nonlinearity, while the fifth-order absorption is equal to zero on resonance experiencing nonzero fifth-order dispersion. The third-order absorption and dispersion enhance in the presence of Fano interference. Yet, the fifth-order susceptibility experiences zero absorption with enhanced nonlinear dispersion at ${\delta }_{p}=0$. Therefore, the quantum interference has no significant effect on optical properties of the third-order susceptibility, whereas it has great impact on the fifth-order susceptibility. When the dephasing rates ${\gamma }_{id}$ become large enough, the impact of tunneling induced interference on the third-order susceptibility becomes less important. In this case, the third-order absorption becomes large on zero probe field detuning. However, increasing the dephasing rate leads to enhanced fifth-order dispersion with no absorption. In Fig. 3, we display the behavior of nonlinear susceptibilities versus the probe detuning in the absence (solid line) and presence (dashed line) of Fano interference for the non-resonant coupling field. We find that (a) the third-order absorption in the absence (solid line) and presence (dashed line) of Fano interference has a zero value at ${\delta }_{p}=0$. However, away from resonance condition ${\delta }_{p}\ne 0$, its value increases in presence of the Fano interference. The cross-Kerr nonlinear coefficient also increases when we consider the Fano interference effect in the system (Fig. 3(b)). Therefore, the Fano interference enhances the cross-Kerr nonlinear coefficient accompanied by vanishing nonlinear absorption for the non-resonance coupling light. The fifth-order probe susceptibility behaves similarly with the third-order susceptibility, as illustrated in Fig. 3(c,d). Figure 4(a,b) show the amplitude and phase modulations of total susceptibility versus x and y in the absence of Fano interference at ${\delta }_{c}=-\,6\gamma$. Here, we have relatively small amplitude and large phase modulations for total probe susceptibility. Therefore, we expect that a portion of probe energy is gathered in the zero-order component with other portions gathering in higher orders directions. The impact of Fano interference on amplitude and phase modulations for total susceptibility is displayed in Fig. 5. One can see that due to the Fano interference effect, the efficiency of amplitude and phase modulations are modified. The amplitude and phase modulations are respectively large due to the presence of Fano interference. The dephasing rates ${\gamma }_{id}$ become small physically in the presence of the Fano interference. Consequently, the tunneling interference becomes very important, leading to the increase of dispersion (third- and fifth- orders) as well as the phase diffraction patterns. Therefore, one expects that the efficiencies of Fraunhofer diffractions are improved when both the third- and fifth-order susceptibilities are taking into account. Therefore, phase modulation is possible in the system due to both third-order and fifth order nonlinear components of probe susceptibility. Also, the medium becomes nearly transparent for the probe light due to the two-dimensional standing-wave pattern of the coupling field. In Fig. 6, we show the normalized Fraunhofer diffraction patterns versus $\sin \,{\theta }_{x}$ and $\sin \,{\theta }_{y}$ using total (a), first-order (b), third-order (c) and fifth-order (d) susceptibilities in the absence of Fano interference for ${\delta }_{c}=0$. For the total probe susceptibility (a), some portion of probe energy is accumulated in the zero-order while other portions of probe energy backlog in higher orders directions. The role of different components of the probe susceptibily on Fraunhofer diffraction pattern are displayed Fig. 6(b–d). When only the first- order (b) and third-order (c) orders susceptibilities are considered, most of the probe energy gathers in the zero-order while all higher-order directions are zero. One can see from Fig. 6(d) that the fifth-order susceptibility (d) causes the transferring of probe energy to the higher-order directions. As a result, the main nonlinear mechanism contributed to transferring the probe energy from zero-order to high order-directions stems from the fifth-order susceptibility. These treatments can be interpreted by studying the absorption and dispersion properties of the medium for the third and fifth orders of susceptibility, which was presented in Fig. 2. The third-order susceptibility involves strong absorption with small dispersion, while the fifth-order of susceptibility shows approximately vanishing absorption and strong dispersion at ${\delta }_{p}=0$.

**Figure 7:**
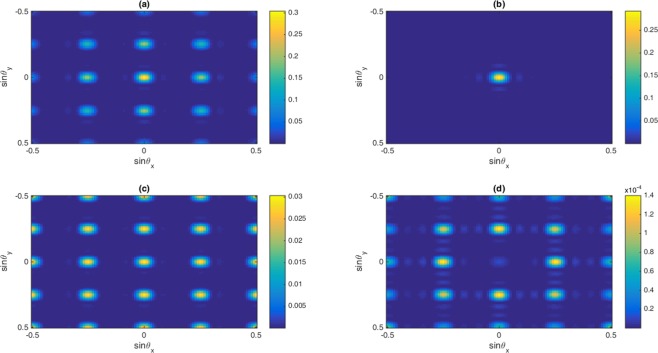
The Normalized Fraunhofer diffraction intensity versus $$\sin \,{\theta }_{x}$$ and $$\sin \,{\theta }_{y}$$ in the presence of Fano interference for (**a**) total susceptibility, (**b**) first-, (**c**) third- and (**d**) fifth-order of susceptibility. The selected parameters are $${\Omega }_{c}=0.65\gamma ,{\gamma }_{2d}=0.32\gamma ,{\gamma }_{3d}=0.38\gamma ,{\gamma }_{2l}=0.84\gamma ,\,{\gamma }_{3l}=1\gamma ,\,{\gamma }_{4l}=0.19\gamma ,{\gamma }_{4d}=0.12\gamma ,$$$${\delta }_{c}=0,\,\delta =2\gamma $$. Our calculation is based on MATLA R2014b software. https://www.mathworks.com/.

In Fig. 7, we display two-dimensional Fraunhofer diffraction patterns for total (a), first-order (b), third-order (c) and fifth-order (d) susceptibilities in the presence of Fano interference at ${\delta }_{c}=0$. We find that the efficiency of diffraction patterns enhances for total and different orders probe susceptibilities. The Fano interference enhances the cross-Kerr nonlinear coefficient and therefore nonzero higher orders diffractions appear in the Fraunhofer diffraction pattern. Yet, due to strong third-order absorption, most of the probe energy is placed in the zero-order direction. Meanwhile, for fifth-order probe susceptibility, we have phase grating in the system due to suppressing the fifth-order absorption and enhancing of dispersion. Hence, it is realized that the Fano interference can improve the efficiency of amplitude grating for the third-order susceptibility and phase grating for fifth-order susceptibility at ${\delta }_{c}=0$. However, the behavior of Fraunhofer diffraction for total susceptibility shows that the main mechanism for adjusting the diffraction patterns comes from the contribution of the first-order and third-order susceptibilities.

**Figure 8:**
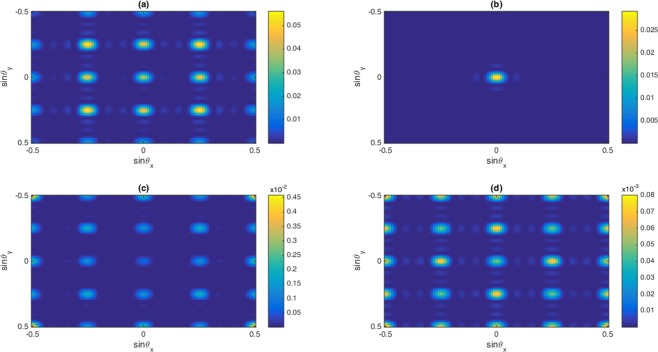
The Normalized Fraunhofer diffraction intensity versus $\sin \,{\theta }_{x}$ and $\sin \,{\theta }_{y}$ in the absence of Fano interference for (**a**) total susceptibility, (**b**) first-, (**c**) third- and (**d**) fifth-order of susceptibility. The selected parameters are ${\Omega }_{c}=0.65\gamma ,{\gamma }_{2d}=0.32\gamma ,{\gamma }_{3d}=0.38\gamma ,{\gamma }_{2l}=0.84\gamma ,\,{\gamma }_{3l}=1\gamma ,\,{\gamma }_{4l}=0.19\gamma ,{\gamma }_{4d}=0.12\gamma ,$ ${\delta }_{c}=-6\gamma ,\,\delta =2\gamma$. Our calculation is based on MATLA R2014b software. https://www.mathworks.com/.

In Fig. 8, we show the two-dimensional Fraunhofer diffraction pattern for total (a), first-order (b), third-order (c) and fifth-order (d) susceptibilities in the absence of Fano interference and ${\delta }_{c}=-\,6\gamma$. Figure 8(a) reveals that some portion of the probe energy gathers in zero-order and some portion of energy gathers in higher orders. In this case, we have approximately identical amplitude and phase modulations. Therefore, the efficiencies of Fraunhofer diffraction for the zero-order or higher orders directions are identical. However, the diffraction efficiency due to third-order susceptibility is higher than diffraction efficiency due to the fifth-order susceptibility.

**Figure 9:**
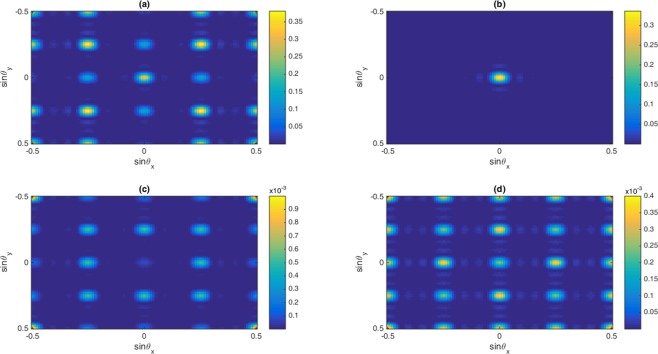
The Normalized Fraunhofer diffraction intensity versus $$\sin \,{\theta }_{x}$$ and $$\sin \,{\theta }_{y}$$ in the presence of Fano interference for (**a**) total susceptibility, (**b**) first-, (**c**) third- and (**d**) fifth-order of susceptibility. The selected parameters are $${\Omega }_{c}=0.65\gamma ,{\gamma }_{2d}=0.32\gamma ,{\gamma }_{3d}=0.38\gamma ,{\gamma }_{2l}=0.84\gamma ,\,{\gamma }_{3l}=1\gamma ,\,{\gamma }_{4l}=0.19\gamma ,{\gamma }_{4d}=0.12\gamma ,$$$${\delta }_{c}=-6\gamma ,\,\delta =2\gamma $$. Our calculation is based on MATLA R2014b software. https://www.mathworks.com/.

Finally, the two-dimensional Fraunhofer diffraction patterns are displayed in Fig. 9 for total (a), first-order (b), third-order (c) and fifth-order (d) susceptibilities in the presence of Fano interference when ${\delta }_{c}=-\,6\,\gamma$. We observe that the diffraction efficiency of all components of the probe susceptibility is modified in the presence of Fano interference. In particular, the intensity of zero-order reduces, and high-order increases due to the presence of Fano interference. In this case, some portion of the probe energy diffracts to the high order of diffraction due to the presence of phase modulation. The phase coefficient of the probe light (third-order and fifth-order) becomes physically large and, therefore, the diffraction efficiency and phase modulations increase, respectively.

## Conclusion

We have investigated the two-dimensional electromagnetically induced phase grating in a semiconductor quantum well system via adjusting the third-order and fifth-order susceptibilities. The effect of resonant tunneling on diffraction efficiency of probe light has been studied. Based on the results, the third-order and fifth-order susceptibilities can be manipulated by controlling the strength of Fano interference in off-resonance of coupling light. We find that the ability of grating can be controlled for third-order and fifth-order susceptibilities due to the Fano interference-effect. Also, the energy could transfer from zero-to high orders diffractions when we consider the third-order or fifth-order susceptibilities in different parametric conditions. Thus, phase grating is possible for both third-order and fifth-order susceptibilities by adjusting the detuning of coupling light and in the presence of Fano interference. The results indicate that the phase modulation of the medium is more significant than the amplitude modulation.
